# Supplementary material for: Activation of the insulin receptor by insulin-like growth factor 2
Source: Nat Commun. 2024 Mar 23;15:2609. doi: 10.1038/s41467-024-46990-6 (PMC10960814; doi:10.1038/s41467-024-46990-6)
Supplement: Supplementary file 1 — Supplementary Information [file 41467_2024_46990_MOESM1_ESM.pdf]

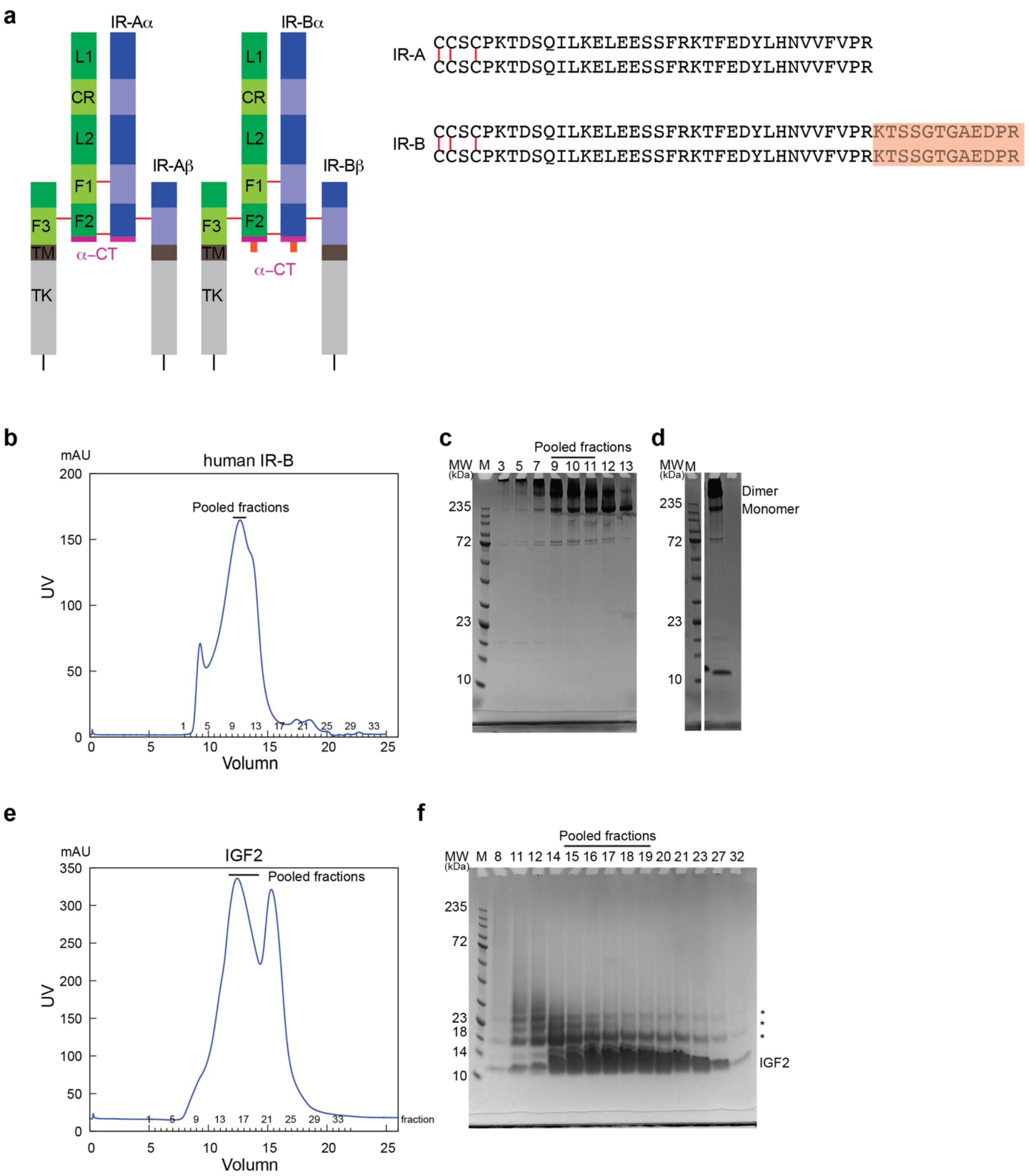

Supplementary Figure 1. Purification of IGF2 and full-length human IR-B

a. Domains of IR-A and IR-B. L1 and L2, leucine rich domains 1 and 2; CR, cysteine rich domain; F1, F2, and F3, fibronectin III (FnIII) domains; α-CT, C-terminal of the alpha subunit; TM, transmembrane domain; TK, tyrosine kinase domain. 12-residue at the C-terminus of IR B α-CT

was noted as orange. Sequences of the C-terminal region of α-chains in IR-A and IR-B. Disulfide bonds are marked in red.

b,c. Purification of human IR-B. A representative size-exclusion chromatography of hIR-B (**b**) and the corresponding sodium dodecyl sulfate polyacrylamide gel electrophoresis (SDS-PAGE) analysis results (**c**).

d. A representative sample for cryo-EM grids preparation.

e,f. Purification of human IGF2. A representative size-exclusion chromatography of IGF2 (**e**) and the corresponding SDS-PAGE analysis results (**f**). * represents improperly folded IGF2 ligand.

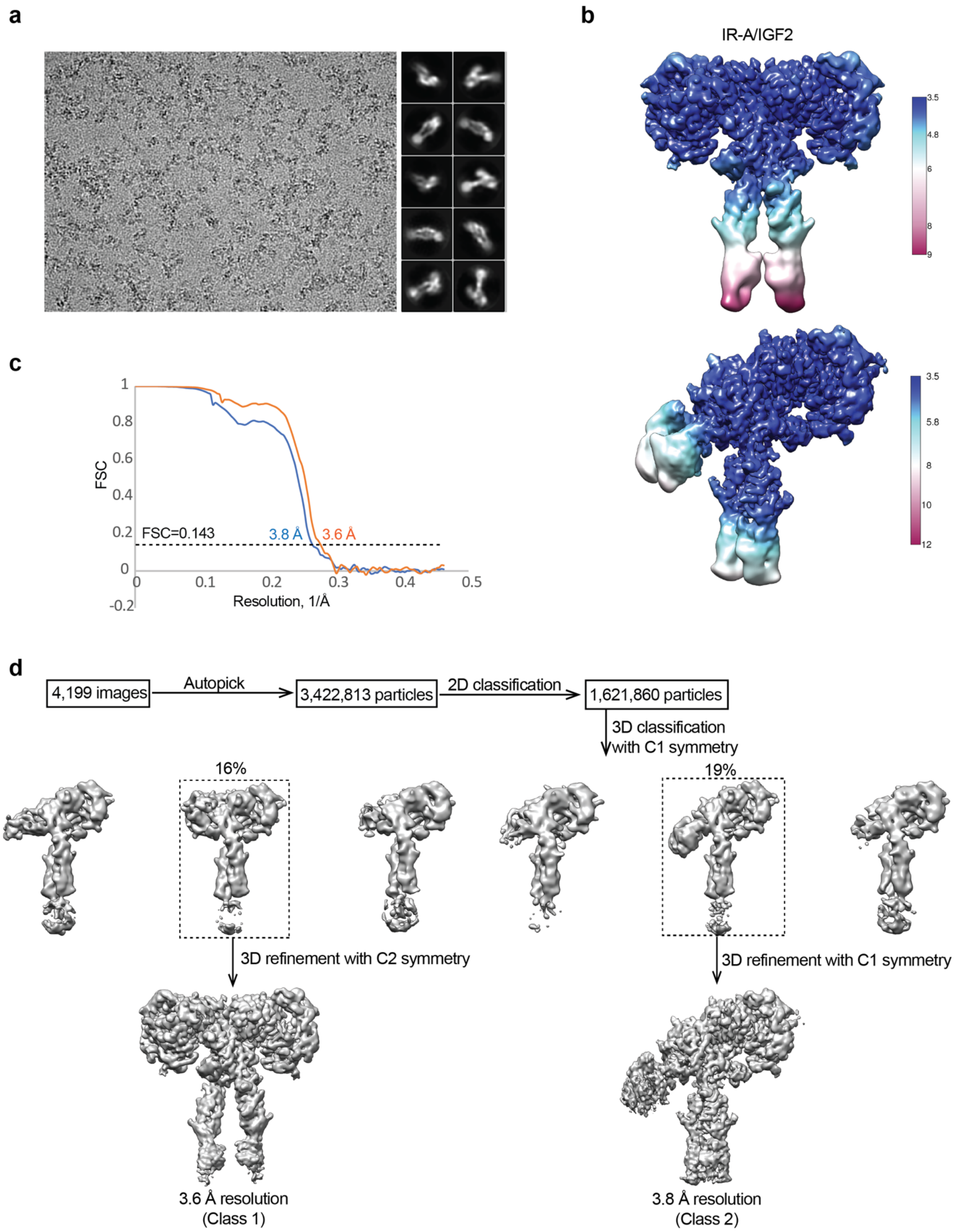

Supplementary Figure 2. Cryo-EM analysis of the IR-A/IGF2 complex.

a. A representative electron micrograph and 2D class averages of the IR-A/IGF2 complex.

b. Unsharpened cryo-EM map colored by local resolution.

c. The gold-standard Fourier shell correlation curve (FSC) for the cryo-EM map shown in **Fig.1c,d** and **Supplementary Fig.4**.

d. Flowchart of cryo-EM data processing.

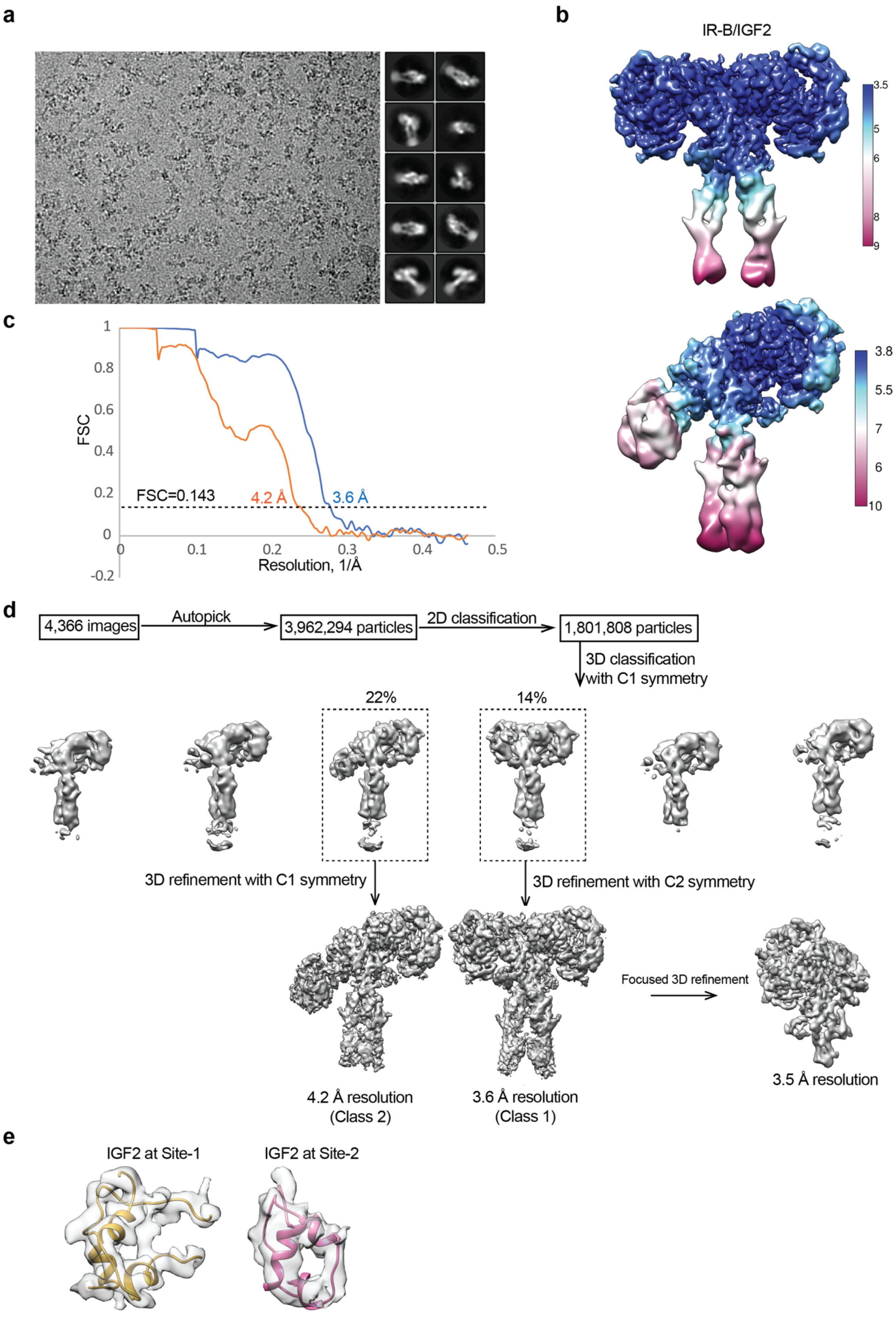
a
b
IR-B/IGF2
3.5
5
6
8
9
3.8
5.5
7
6
10
c
FSC
Resolution, 1/Å
FSC=0.143
4.2 Å
3.6 Å
1
0.8
0.6
0.4
0.2
0
-0.2
0
0.1
0.2
0.3
0.4
0.5
d
4,366 images
Autopick
3,962,294 particles
2D classification
1,801,808 particles
3D classification
with C1 symmetry
22%
14%
3D refinement with C1 symmetry
3D refinement with C2 symmetry
Focused 3D refinement
4.2 Å resolution
(Class 2)
3.6 Å resolution
(Class 1)
3.5 Å resolution
e
IGF2 at Site-1
IGF2 at Site-2

Supplementary Figure 3. Cryo-EM analysis of the IR-B/IGF2 complex.

a. A representative electron micrograph and 2D class averages of the IR-B/IGF2 complex.

b. Unsharpened cryo-EM map colored by local resolution.

c. The gold-standard Fourier shell correlation curve (FSC) for the cryo-EM map shown in **Fig. 1**.

d. Flowchart of cryo-EM data processing.

e. Cryo-EM density of IGF2 at site-1 (left, yellow) and site-2 (right, purple).

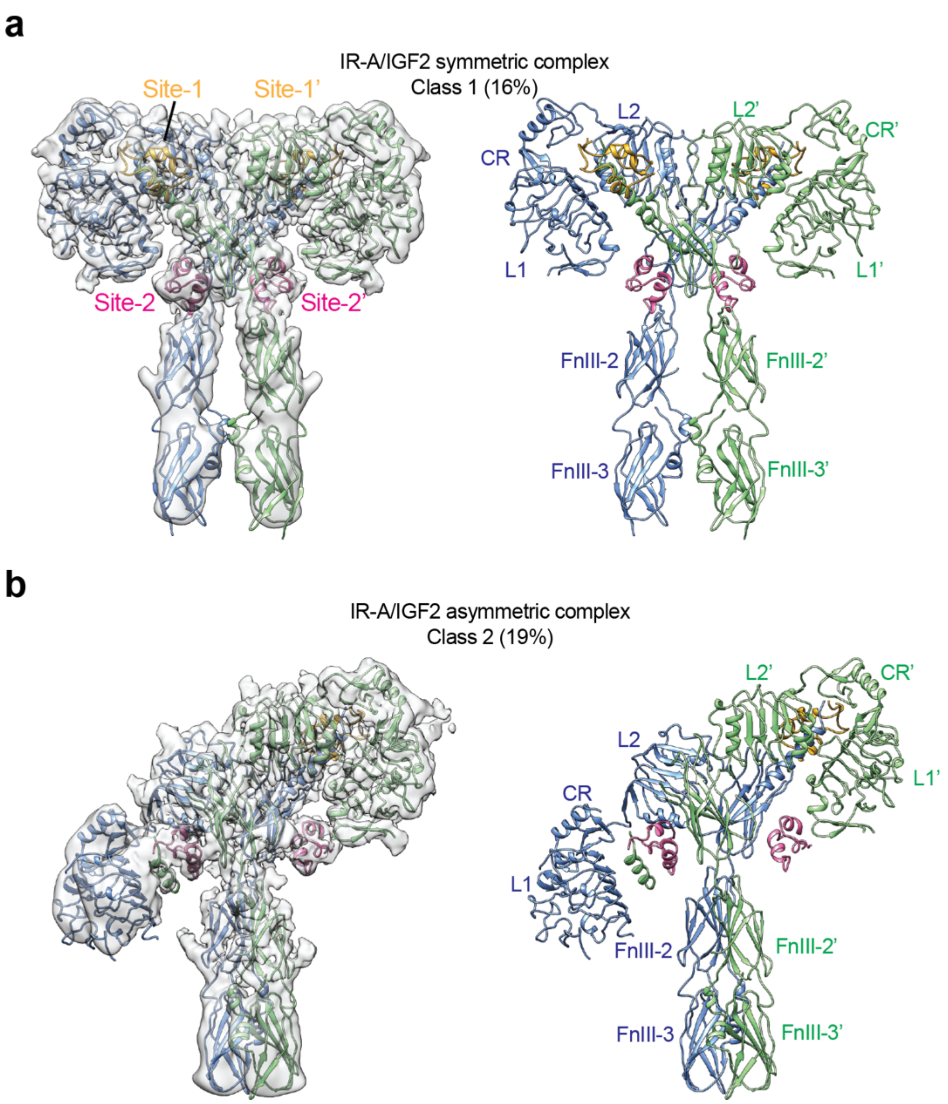

Supplementary Figure 4. Overall structures of IR-A/IGF2 complex.

a. 3D reconstruction of IR-A/IGF2 complex in symmetric conformation fitted into a cryo-EM map at 3.6 Å resolution (left). Ribbon representation of the symmetric IR-A/IGF2 complex (right).

b. 3D reconstruction of IR-A/IGF2 complex in asymmetric conformation fitted into a cryo-EM map at 3.8 Å resolution (left). Ribbon representation of the asymmetric IR-A/IGF2 complex (right).

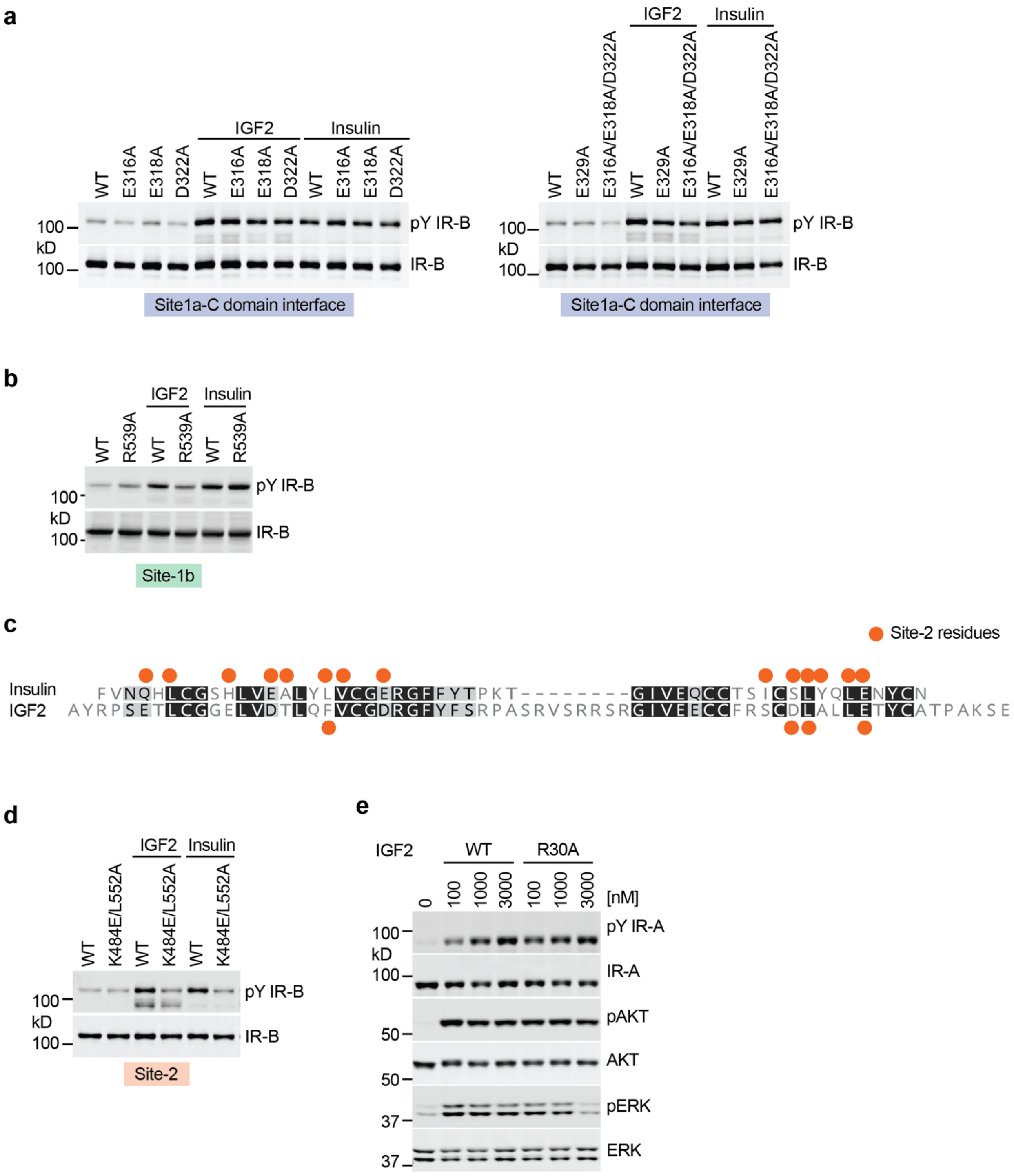

Supplementary Figure 5. IGF2- and insulin-induced IR activation.

a. Autophosphorylation of IR-B (pY IR) by 100 nM IGF2 and 10 nM insulin for 10 min in IR/IGF1R double knockout 293FT cells expressing IR-B WT or the indicated IR-B mutants. Quantification is shown in **Fig. 2c**. Source data are provided as a Source Data file.

b. Autophosphorylation of IR-B (pY IR) by 100 nM IGF2 and 10 nM insulin for 10 min in IR/IGF1R double knockout 293FT cells expressing IR-B WT or IR-B R539A. Quantification is shown in **Fig. 2d**. Source data are provided as a Source Data file.

c. Sequence alignment of human insulin and IGF2. Key site-2 residues are marked in orange.

d. Autophosphorylation of IR-B (pY IR) by 100 nM IGF2 and 10 nM insulin for 10 min in IR/IGF1R double knockout 293FT cells expressing IR WT or IR K484E/L552A. Quantification is shown in **Fig. 2g**. Source data are provided as a Source Data file.

e. IR signaling in IR and IGF1R double knockout preadipocytes expressing only mouse IR-A (DKO-IR-A) treated with the indicated ligands for 10 min. Quantification is shown in **Fig. 4d**. Source data are provided as a Source Data file.

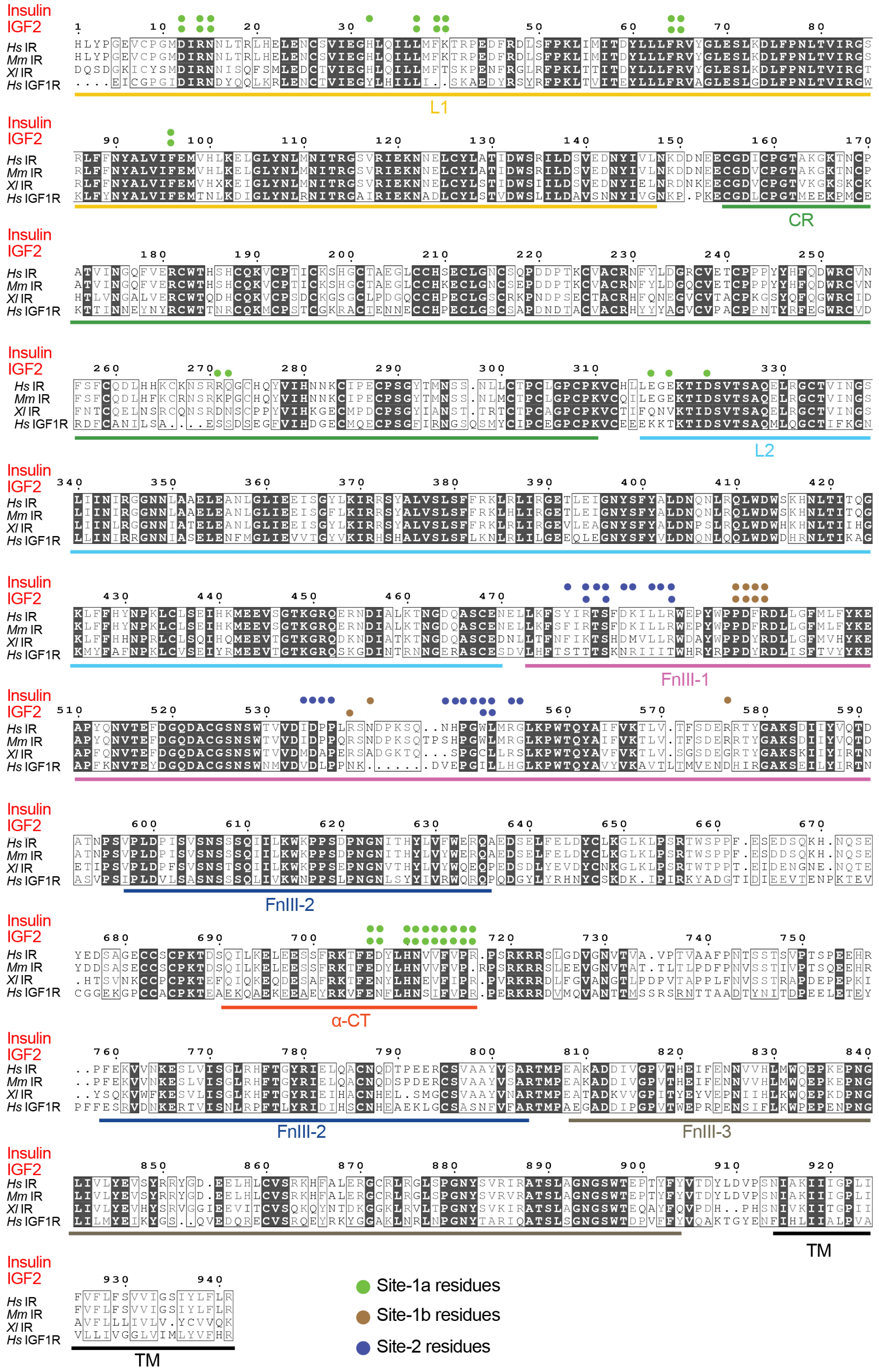

Supplementary Figure 6. Sequence alignment of IR proteins from human (*Hs*), mouse (*Mm*), xenopus (*Xl*), and human IGF1R. Insulin and IGF2-binding residues are marked in colors.

	pY/IR	pAKT/AKT	pERK/ERK
WT	99.96±0.92 (n=16)	104.6±3.21 (n=15)	99.51±1.61 (n=15)
E12A	21.1±1.97 (n=4)****	38.7±2.85 (n=4)****	47.56±3.78 (n=4)****
V43E	15.77±0.96 (n=5)****	6.8±1.42 (n=4)****	25.08±10.72 (n=4)****
F19A/L53A	146.8±22.43 (n=5)	64.3±4.89 (n=4)***	65.09±4.22 (n=4)**
R37A	85.33±7.3 (n=4)	59.92±3.5 (n=4)****	61.63±8.15 (n=4)**
R38A	90.41±3.87 (n=6)	83.17±6.58 (n=6)*	76.22±5.28 (n=6)
R37A/R38A	116.8±16.57 (n=7)	60.58±2.6 (n=6)****	60.53±7.61 (n=6)***
E12A/R37A/R38A	19.11±0.77 (n=4)****	43.68±4.18 (n=4)****	52.1±3.25 (n=4)****
R30A	190.5±13.65 (n=4)****	126.6±11.93 (n=4)*	116.6±2.13 (n=4)

IGF2 mutant activity to IR-B is expressed as a percent of IGF2 WT activity for 10 min (mean±s.e.m.).
*p<0.05; **p<0.01; ***p<0.001, and ****p<0.0001

Supplementary Figure 7. Relative activity of IGF2 WT and the IGF2 mutants for IR-B. Source data are provided as a Source Data file.

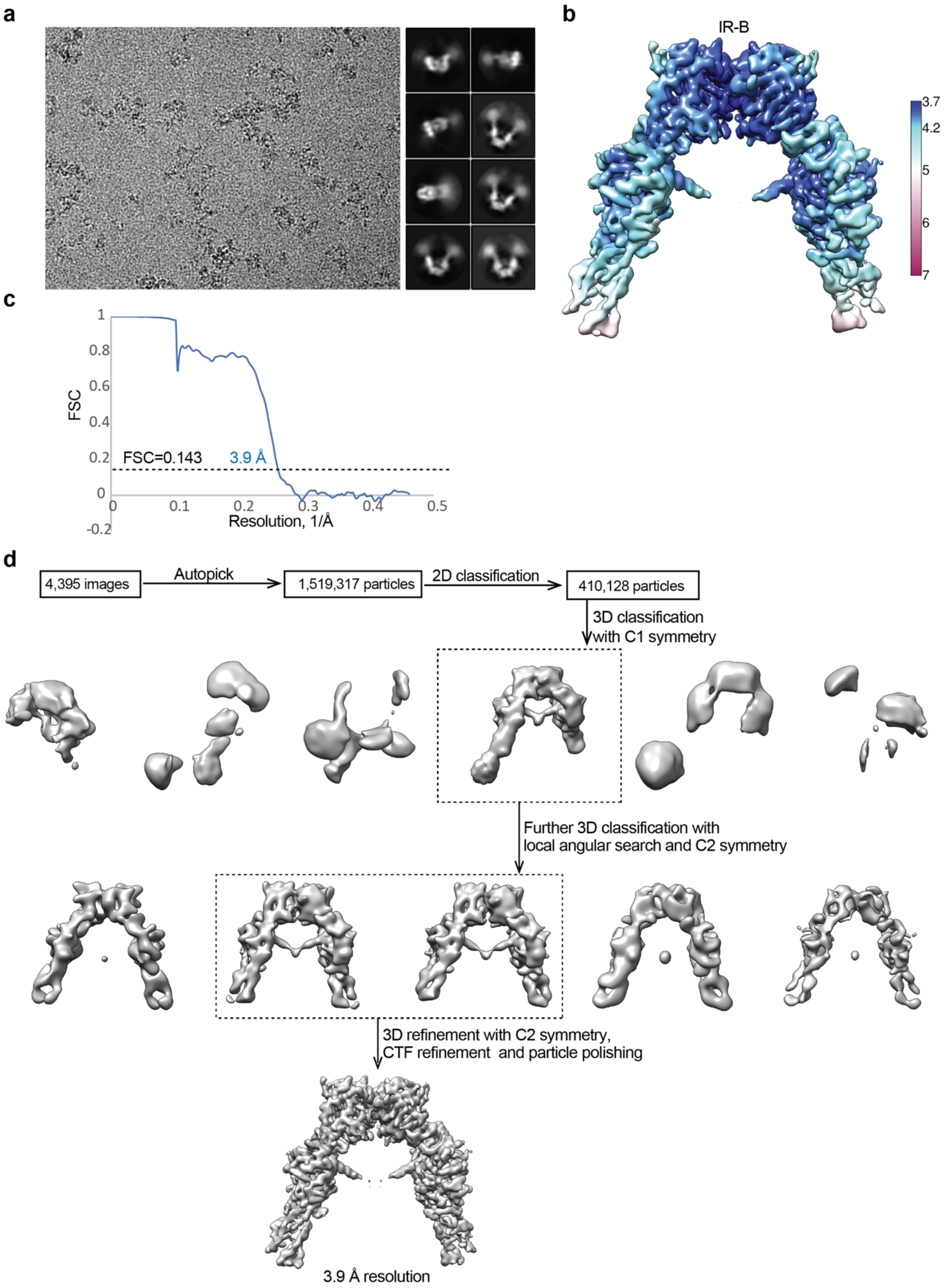

Supplementary Figure 8. Cryo-EM analysis of the ligand free IR-B.

a. A representative electron micrograph and 2D class averages of IR-B.

b. Unsharpened cryo-EM map colored by local resolution.

c. The gold-standard Fourier shell correlation curve (FSC) for the cryo-EM map shown in **Fig. 5**.

d. Flowchart of cryo-EM data processing.

Supplementary Table 1. Sequences of primers

IR E316A	Forward	CCACCTCCTAgcaGGCGAGAAGA
	Reverse	CACACCTTGGGACAGGGA
IR E318A	Forward	CTAGAAGGCGcGAAGACCATCGAC
	Reverse	GAGGTGGCACACCTTGGG
IR D322A	Forward	AAGACCATCGcCTCGGTGACG
	Reverse	CTCGCCTTCTAGGAGGTG
IR E316A/E318A/D322A	Forward	gaccatcgccTCGGTGACGTCTGCCCAG
	Reverse	ttcgcgcctgcTAGGAGGTGGCACACCTTG
IR R539A	Forward	CCCACCCCTGgcGTCCAACGAC
	Reverse	TCAATGTCTACCACCGTC
IR N594A	Forward	AGATGCCACCgcCCCCTCTGTG
	Reverse	GTCTGGACATAAATGATGTC
IR N594E	Forward	AGATGCCACCgagCCCTCTGTGC
	Reverse	GTCTGGACATAAATGATGTCACTCTTG
IR N594R	Forward	AGATGCCACCcgCCCCTCTGTG
	Reverse	GTCTGGACATAAATGATGTC
IR E329A	Forward	TCTGCCCAGGcGCTCCGAGGA
	Reverse	CGTCACCGAGTCGATGGTC
IGF2 R37AR38A	Forward	TCGCGTTAGCgctgccAGTCGTGGAATTGTGG
	Reverse	CTTGCCGGGCGCGAAAAA
IGF2 F19A	Forward	CACCCTTCAGgcaGTATGTGGCG
	Reverse	TCTACCAATTCACCACCG
IGF2 L53A	Forward	CTCCTGTGACgcgGCGCTGTTAG
	Reverse	CGAAAGCAGCACTCCTCC
IGF2 V43E	Forward	CGTGGAATTGaGGAGGAGTGCTGCTTTCG

	Reverse	ACTGCGGCGGCTAACGCG
IGF2 E12A	Forward	ATGCGGTGGTgctTTGGTAGACAC
	Reverse	AATGTTTCCGATGGACGATATG
IGF2 R30A	Forward	TTATTTTTCGgcgCCGGCAAGTCGCGTTAG
	Reverse	AATCCACGGTCGCCACAT
IGF2 R38A	Forward	CGTTAGCCGCgctAGTCGTGGAATTGTGGAGGAGTGC
	Reverse	CGACTTGCCGGGCGCGAA
IGF2 E12H	Forward	ATGCGGTGGTcacTTGGTAGACAC
	Reverse	AATGTTTCCGATGGACGATATG
IGF2 E12Q	Forward	ATGCGGTGGTcagTTGGTAGACAC
	Reverse	AATGTTTCCGATGGACGATATG
IGF2 R37A	Forward	TCGCGTTAGCgcgCGCAGTCGTGGAATTG
	Reverse	CTTGCCGGGCGCGAAAAA
IGF2 A54Y	Forward	CTGTGACTTGtatCTGTTAGAGACATATTGCGC
	Reverse	GAGCGAAAGCAGCACTCC
IGF2 F19L	Forward	CACCCTTCAGttaGTATGTGGCG
	Reverse	TCTACCAATTCACCACCG
IGF2 D15E	Forward	TGAATTGGTAgagACCCTTCAGT
	Reverse	CCACCGCATAATGTTTCC
IGF2 R40A	Forward	CCGCCGCAGTgcaGGAATTGTGGAGGAGTG
	Reverse	CTAACGCGACTTGCCGGG
