# Supplementary material for: Activation of the insulin receptor by insulin-like growth factor 2
Source: Nat Commun. 2024 Mar 23;15:2609. doi: 10.1038/s41467-024-46990-6 (PMC10960814; doi:10.1038/s41467-024-46990-6)
Supplement: Supplementary file 2 — Reporting Summary [file 41467_2024_46990_MOESM2_ESM.pdf]

nature portfolio

Corresponding author(s): Eunhee Choi, Xiao-chen Bai

Last updated by author(s): 2024 3 4

Reporting Summary

Nature Portfolio wishes to improve the reproducibility of the work that we publish. This form provides structure for consistency and transparency in reporting. For further information on Nature Portfolio policies, see our Editorial Policies and the Editorial Policy Checklist.

Statistics

For all statistical analyses, confirm that the following items are present in the figure legend, table legend, main text, or Methods section.

n/a	Confirmed	
☐	☒	The exact sample size (n) for each experimental group/condition, given as a discrete number and unit of measurement
☐	☒	A statement on whether measurements were taken from distinct samples or whether the same sample was measured repeatedly
☐	☒	The statistical test(s) used AND whether they are one- or two-sided<br>*Only common tests should be described solely by name; describe more complex techniques in the Methods section.*
☒	☐	A description of all covariates tested
☒	☐	A description of any assumptions or corrections, such as tests of normality and adjustment for multiple comparisons
☐	☒	A full description of the statistical parameters including central tendency (e.g. means) or other basic estimates (e.g. regression coefficient) AND variation (e.g. standard deviation) or associated estimates of uncertainty (e.g. confidence intervals)
☐	☒	For null hypothesis testing, the test statistic (e.g. F, t, r) with confidence intervals, effect sizes, degrees of freedom and P value noted<br>*Give P values as exact values whenever suitable.*
☒	☐	For Bayesian analysis, information on the choice of priors and Markov chain Monte Carlo settings
☒	☐	For hierarchical and complex designs, identification of the appropriate level for tests and full reporting of outcomes
☒	☐	Estimates of effect sizes (e.g. Cohen's d, Pearson's r), indicating how they were calculated

Our web collection on statistics for biologists contains articles on many of the points above.

Software and code

Policy information about availability of computer code

Data collection	EPU
Data analysis	RELION4<br>MotionCor2<br>GCTF1.06<br>Coot0.8.8<br>PHENIX1.17<br>Chimera1.17<br>MolProbity 4.5<br>SerialEM 3.8<br>All software used is commercially or publicly available and described in the Methods section. Data displayed in graphs were analyzed using GraphPad Prism 10 (GraphPadSoftware Inc.)

For manuscripts utilizing custom algorithms or software that are central to the research but not yet described in published literature, software must be made available to editors and reviewers. We strongly encourage code deposition in a community repository (e.g. GitHub). See the Nature Portfolio guidelines for submitting code & software for further information.

Data

Policy information about availability of data

All manuscripts must include a data availability statement. This statement should provide the following information, where applicable:

- Accession codes, unique identifiers, or web links for publicly available datasets
- A description of any restrictions on data availability
- For clinical datasets or third party data, please ensure that the statement adheres to our policy

All reagents generated in this study are available with a completed Materials Transfer Agreement. All cryo-EM maps and models reported in this work has been deposited into EMDB/PDB database, under the entry ID: EMD-41877 [https://www.ebi.ac.uk/emdb/EMD-41877] (Apo-IR-B), PDB 8U4B [https://www.rcsb.org/structure/unreleased/8U4B] (Apo-IR-B), EMD-41878 [https://www.ebi.ac.uk/emdb/EMD-41878] (Symmetric IR-B/IGF2), PDB 8U4C [https://www.rcsb.org/structure/unreleased/8U4C] (Symmetric IR-B/IGF2), EMD-41880 [https://www.ebi.ac.uk/emdb/EMD-41880] (Asymmetric IR-B/IGF2), PDB 8U4E [https://www.rcsb.org/structure/unreleased/8U4E] (Asymmetric IR-B/IGF2), EMD-43279 [https://www.ebi.ac.uk/emdb/EMD-43279] (Symmetric IR-A/IGF2), PDB 8VJB [https://www.rcsb.org/structure/unreleased/8VJB] (Symmetric IR-A/IGF2), EMD-43280 [https://www.ebi.ac.uk/emdb/EMD-43280] (Asymmetric IR-A/IGF2), and PDB 8VJC [https://www.rcsb.org/structure/unreleased/8VJC] (Asymmetric IR-A/IGF2). Source data are provided with this paper. PDB used in this study are as follows: 1IGL [https://www.rcsb.org/structure/1IGL], 4ZXB [https://www.rcsb.org/structure/4ZXB] and 6PXV [https://www.rcsb.org/structure/6PXV].

Field-specific reporting

Please select the one below that is the best fit for your research. If you are not sure, read the appropriate sections before making your selection.

☒ Life sciences ☐ Behavioural & social sciences ☐ Ecological, evolutionary & environmental sciences

For a reference copy of the document with all sections, see nature.com/documents/nr-reporting-summary-flat.pdf

Life sciences study design

All studies must disclose on these points even when the disclosure is negative.

Sample size	The sample sizes were determined based on our extensive experiences and according to results from pilot experiments. Sample sizes are given in the manuscript.
Data exclusions	Only the particles that showed homogeneous conformation were selected in the final reconstruction.
Replication	Multiple independent repeats were included for related experiments and noted as dots in Figures. Each experiment was performed at least three times to corroborate that data are reproducible.
Randomization	The allocation of samples in this study was performed randomly. The samples were randomly analyzed. For cryo-EM resolution estimation, all particles were randomly split into two groups.
Blinding	The investigators were blinded to group allocation during data collection and analysis.

Reporting for specific materials, systems and methods

We require information from authors about some types of materials, experimental systems and methods used in many studies. Here, indicate whether each material, system or method listed is relevant to your study. If you are not sure if a list item applies to your research, read the appropriate section before selecting a response.

Materials & experimental systems

n/a	Involved in the study	
☐	☒	Antibodies
☐	☒	Eukaryotic cell lines
☒	☐	Palaeontology and archaeology
☐	☒	Animals and other organisms
☒	☐	Human research participants
☒	☐	Clinical data
☒	☐	Dual use research of concern

Methods

n/a	Involved in the study	
☒	☐	ChIP-seq
☒	☐	Flow cytometry
☒	☐	MRI-based neuroimaging

Antibodies

Antibodies used	Anti-IR-pY1150/1151 (1:2000, 19H7, Cell signaling; labeled as pY IR, Cat. #3024)<br>anti-Myc (1:2000; 9E10, Roche; labeled as IR, Cat. #11667149001)<br>anti-IR (1:500; CT3, Santa Cruz, Cat. #sc-57342)

	anti-AKT (WB, 1:2000; 40D4, Cat. #2920)(Cell signaling)<br>anti-pS473 AKT (WB, 1:2000; D9E, Cat. #4060)(Cell signaling)<br>anti-ERK1/2 (WB, 1:2000; L34F12, Cat. #4696)(Cell signaling)<br>anti-pERK1/2 (WB, 1:2000; 197G2, Cat. #4377)(Cell signaling)<br>Anti-rabbit immunoglobulin G (IgG) (H+L) (Dylight 800 conjugates), used at 1:5000(Cell signaling)<br>anti-rabbit immunoglobulin G (IgG) (H+L) (Dylight 800 conjugates, Cat. #5151)(Cell signaling)<br>anti-mouse IgG (H+L) (Dylight 680 conjugates, Cat. #5470) (Cell signaling)
Validation	1. Anti-IR-pY1150/1151 (19H7, Cell signaling) has been validated by Cell signaling by demonstrating immunoblotting on human cell lysates with and without ligand (see website). This antibody was also validated in our previous work (Choi et al.,2016; Choi et al., 2019, Li et al., 2019, and Uchikawa et al., 2019).<br>2. Anti-Myc (9E10, Roche) has been validated by Roche/Sigma by demonstrating "the monoclonal antibody against the c-myc epitope is well characterized and does not cross-react with other cellular proteins". This antibody was also validated in our previous work (Uchikwa et al., 2019, Li et al., 2022, and Wang et al., 2023).<br>3. Anti-AKT (40D4, Cell signaling, labeled as AKT) has been validated by Cell signaling by demonstrating immunoblotting on extracts from NIH/3T3, C6 and COS cells. This antibody was also validated in our previous work (Choi et al., 2016; and Choi et al., 2019).<br>4. Anti-pS473 AKT (D9E, Cell signaling, labeled as pS473 AKT) has been validated by Cell signaling by demonstrating immunoblotting on extracts from PC-3, untreated or treated inhibitor, and NIH3T3 cells, serum starved or PDGF-treated. This antibody was also validated in our previous work (Choi et al., 2016; and Choi et al., 2019).<br>5. Anti-ERK1/2 (L34F12, Cell signaling, labeled as ERK1/2) has been validated by Cell signaling by demonstrating immunoblotting on extracts from NIH/3T3, PC12 and COS cells. This antibody was also validated in our previous work (Choi et al., 2016; and Choi et al., 2019).<br>6. Anti-pERK1/2 (197G2, Cell signaling, labeled as pERK1/2) has been validated by Cell signaling by demonstrating immunoblotting on purified protein or extracts from NIH/3T3 cells treated with UV light and PDGF. This antibody was also validated in our previous work (Choi et al., 2016; and Choi et al., 2019).<br>7. Anti-IR (CT-3, Santa Cruz, labeled as IR) has been validated by Santa Cruz by demonstrating immunoblotting on lysates from NIH/3T3, SW480, MCF7, JAR, MIA PaCa-2 and HepG2. This antibody was also validated in our previous work (Choi et al.,2016; Choi et al., 2019, and Uchikawa et al., 2019).

Eukaryotic cell lines

Policy information about cell lines

Cell line source(s)	293FT (Invitrogen, R70007), FreeStyleTM 293-F (Invitrogen, R79007), Sf9 (Gibco, 11496015), HeLa Tet-on (Takara Bio), and C2C12 (ATCC, CRL-1772) cell lines were purchased from company. Brown preadipocytes were obtained from Dr. Ronald Kahn.
Authentication	We freshly purchased cell lines from company and did not perform the additional authentication process.
Mycoplasma contamination	Negative for mycoplasma
Commonly misidentified lines<br>(See ICLAC register)	No commonly misidentified cell lines were used.

Animals and other organisms

Policy information about studies involving animals; ARRIVE guidelines recommended for reporting animal research

Laboratory animals	C57BL/6J (Jackson laboratory, 000664)
Wild animals	No wild animals were used in this study.
Field-collected samples	No field-collected samples were used in this study.
Ethics oversight	Animal work described in this manuscript has been approved and conducted under the oversight of the Columbia University Institutional Animal Care and Use Committee (AABT9653).

Note that full information on the approval of the study protocol must also be provided in the manuscript.
